# Investigation of tube replacement cases of percutaneous transesophageal gastro-tubing in cancer patients

**DOI:** 10.1007/s11604-025-01770-0

**Published:** 2025-03-29

**Authors:** Rakuhei Nakama, Miyuki Sone, Shunsuke Sugawara, Chihiro Itou, Shintaro Kimura, Mizuki Ozawa, Takumi Oshima, Sho Murakami, Masahiko Kusumoto

**Affiliations:** Department of Diagnostic Radiology, National Cancer Center Hospital, 5-1-1 Tsukiji, Chuo-ku, Tokyo, 104-0045 Japan

**Keywords:** Percutaneous transesophageal gastro-tubing, Tube replacement, Palliative care, Oncology, Gastrostomy

## Abstract

**Purpose:**

Percutaneous transesophageal gastro-tubing (PTEG) is an interventional radiology technique used for enteral feeding, or drainage in malignant bowel syndrome cases, serving as an alternative to percutaneous gastrostomy. Despite its safety and effectiveness in improving quality of life, comprehensive studies on PTEG tube replacement are limited. This study aimed to investigate the cases of PTEG tube replacement.

**Material and methods:**

This single-center retrospective cohort study was conducted at the National Cancer Center Hospital, Tokyo, Japan. Data were collected from patients who underwent PTEG for malignant bowel obstruction or enteral feeding and then required tube replacement between January 1, 2014 and December 31, 2023. Patient characteristics, duration of tube indwelling, PTEG tube tip position, causes of replacement, and whether dilation or re-PTEG was performed during replacement were analyzed, excluding patients who were transferred or deceased before the initial replacement. Statistical analyses were performed using the Chi-square, Fisher's exact, and Mann–Whitney U tests, with significance set at P < 0.05.

**Results:**

Of 236 patients who underwent PTEG, 56 required an initial tube replacement. The mean age was 55 years, with 51.8 % of patients predominantly male. The primary indication for PTEG was decompression (52 patients). The median tube indwelling duration was 31 days, with the tube tip positioned in the gastric or duodenal in 64.3 % of cases. The most frequent reason for common replacement procedures, performed in 44 patients, was tube dysfunction. Replacement due to accidental removal in 12 patients led to higher rates of dilation or re-PTEG. The duration after accidental removal significantly affected the necessity for dilation or re-PTEG.

**Conclusion:**

This study on initial PTEG tube replacement in cancer patients indicated that tube dysfunction is the primary reason for replacement, and accidental removal is more likely to require dilation or re-PTEG.

## Introduction

Percutaneous transesophageal gastro-tubing (PTEG) is an interventional radiology technique developed in Japan as an alternative to percutaneous gastrostomy for enteral feeding, or drainage in cases of malignant bowel syndrome [1]. Unlike nasogastric tubes (NGT), PTEG tubes bypass the nasal cavity or pharynx, reducing the discomfort associated with indwelling tubes. Notably, a randomized controlled trial demonstrated the superiority of PTEG over NGT in terms of the quality of life [2]. Recently, reports have shown that the tip of the PTEG tube can be placed distal to the stomach and duodenum, enabling effective nutrition and decompression in various situations [3, 4]. In the future, PTEG is anticipated to become a widespread method for nutrition or decompression in patients contraindicated for gastrostomy.

Our previous nationwide database study indicated that PTEG is a generally safe procedure, with few complications requiring interventions such as embolization, percutaneous drainage, or surgery[5]. However, the study revealed that some patients required early tube replacement within 2 weeks after PTEG. As PTEG tubes are generally replaced approximately every 3 to 6 months, early replacement indicates some type of tube troubles. Several reports have highlighted issues with PTEG tubes [2, 6–9], underscoring the importance of appropriate tube management for safely using PTEG.

Despite this background, detailed studies of the challenges of managing PTEG tubes are lacking. Comprehensive assessments of PTEG usage challenges, such as patency and replacement frequency, are essential for its broader application. Understanding how long tubes can function reliably and determining optimal replacement intervals are key considerations for facilitating widespread adoption, especially in palliative and home care settings.

To address this knowledge gap, we aimed to analyze and investigate our cases of PTEG tube replacement.

## Materials and methods

This single-center retrospective cohort study was conducted at the National Cancer Center Hospital, Tokyo, Japan. We analyzed data from patients who underwent PTEG for malignant bowel obstruction or enteral feeding and required tube replacement between January 1, 2014 and December 31, 2023. The study focused on the initial tube replacements for PTEG and extracted data from electronic medical records, including patient characteristics (age, sex, indications for PTEG, and primary cancer site), tube tip position (gastric/duodenal or jejunal), duration of tube indwelling, complications related to replacement, causes of replacement, and whether dilation or re-PTEG was required during the replacement.

We categorized the data into two groups based on differences in tube tip position or causes of replacement: gastric/duodenal group versus jejunal group and common replacement procedure group versus replacement due to accidental removal group. We assessed the percentage of dilation and re-PTEG for replacement due to accidental removal group according to the duration after removal.

Patients who were transferred to another hospital or who died before the initial tube replacement were excluded from this study.

This study was approved by the Institutional Review Board of the National Cancer Center (approval number: 2018-049), and the requirement for informed consent was waived due to data anonymity.

### PTEG procedure and selection of tube tip position

PTEG procedure was executed following the previous report [1]. A rupture-free balloon (RFB) catheter was introduced transnasally. Under either ultrasonography or fluoroscopy guidance, the inflated RFB underwent percutaneous puncture. Subsequently, a guidewire was advanced through the RFB, and navigated into the esophagus-stomach to create the route. A dilation sheath was used to dilate the route. Lastly, a drainage or feeding tube was inserted via that sheath, and the tube tip position was selected according to the following principle.

- 1) If a nasogastric tube or long tube (jejunal tube) had been inserted, the tip of the PTEG tube was placed in the same position.
- 2) In the absence of a nasogastric tube, the tip position of the PTEG tube was positioned within the stomach.

### PTEG tube replacement procedure

The common tube replacement was performed over-the-wire under fluoroscopy. In the replacement due to accidental removal, the guidewire was inserted through the existing tube tract (fistula) under fluoroscopy, and a new tube was then inserted over-the-wire. When it was difficult, the tract was dilated using a dilator, or PTEG was reperformed (i.e., re-PTEG).

### Definitions

Tube patency was determined based on a comprehensive assessment of information such as whether contrast media or normal saline could be injected through the tube without resistance and whether the tube appeared to be obstructed (residue in the tube, kink in the tube observed under fluoroscopy) based on the description in the electronic medical record and the interventional radiology report.

Regarding the causes of replacement, "tube dysfunction" referred to cases where the tube was patent but could not be effectively drained. Specifically, in cases where the tube tip is in the initial placement position but there are objective findings of inadequate drainage, such as a dilated bowel on CT or abdominal x-ray. "Simple tube malposition" was defined as a condition where drainage is not effective due to tube malposition resulting from, among other things, accidental extraction. "Tube type change" was defined as situations where although the tube was patent and drainage was effective, the tube was replaced for various reasons, such as changing from gastric tube to W-ED tube and adjusting the tube size (e.g., due to pain at the puncture site).

### Statistical analysis

Categorical variables were evaluated using the Chi-square test, and Fisher's exact test was applied for categories comprising fewer than ten individuals. The Mann–Whitney U test compared the median values of continuous variables, such as the duration of tube indwelling between the gastric/duodenal group versus the jejunal group and between common replacement procedures and replacement due to accidental removals. Results were presented as percentages. Statistical significance was set at P < 0.05, with all reported P-values being two-tailed. Statistical analyses were conducted using STATA (version 18.0; StataCorp LLC, 2023, College Station, TX, USA).

## Results

Between January 2014 and December 2023, a total of 236 patients underwent PTEG. Among these, 56 patients (23.7 %) who required initial tube replacement were included in this study. All tube replacements were performed under fluoroscopic guidance using guidewires.

Table 1 summarizes the characteristics of the patients who underwent tube replacement with PTEG. The mean age of the patients was 55 years, and the majority were men (51.8%). The primary indications for PTEG were decompression in 52 patients and feeding in four patients. In descending order of frequency, the most common primary cancer sites were the stomach, colon, and pancreas. The median duration of tube indwelling was 31 days. The tube tip was positioned predominantly within gastric/duodenal in 36 cases (64.3%). Common replacement procedures were performed in 44 patients, whereas replacement due to accidental removal occurred in 12 patients. The most frequent reason for common replacement procedures was tube dysfunction (26 cases, 46.4%), followed by tube occlusion and tube type change. Only one procedure-related complication (cellulitis) was observed.

**Table 1:** Patient characteristics, tube indwelling duration, PTEG tube tip position, replacement causes, and whether dilation or re-PTEG was required during PTEG tube replacement

Characteristic	Total patients (n = 56)
Age, years, mean ± SD	55±15
Sex, male, n (%)	29 (51.8)
Indication of PTEG, n (%)	
Decompression	52 (92.9)
Feeding	4 (7.1)
Primary Cancer Site, n (%)	
Gastric	16 (28.6)
Colon	10 (17.6)
Pancreas	9 (16.1)
Other	20 (35.7)
Median duration of tube indwelling, day (IQR)	31 (10–66)
The tube tip position, n (%)	
Gastric / duodenal	36 (64.3)
Jejunal	20 (35.7)
The causes of replacement, n (%)	
Common replacement procedures, n (%)	44 (78.6)
Tube dysfunction	23 (41.1)
Tube occlusion	6 (10.7)
Tube type change	5 (8.9)
Infection of puncture site	4 (7.1)
Simple tube malposition	3 (5.4)
Others	3 (5.4)
Replacement due to accidental removal, n (%)	12 (21.4)
Dilation, n (%)	12 (21.4)
Re-PTEG, n (%)	2 (3.6)
Complications, n (%)	1 (1.8)

**Fig. 1:**
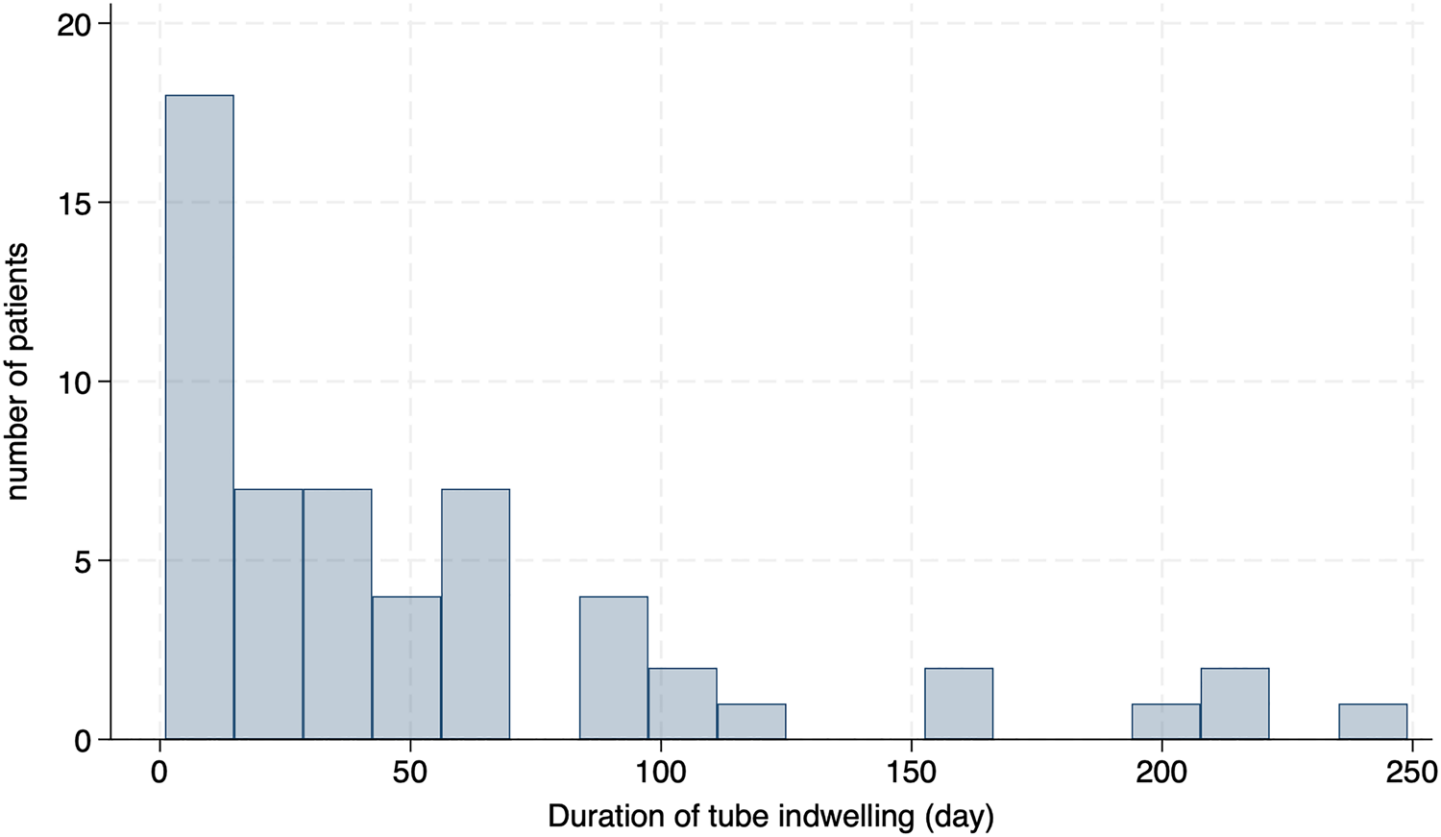
The histogram of the duration of PTEG tube indwelling

Figure 1 presents a histogram depicting the duration of PTEG tube indwelling. In this study, 18 cases (32.1%) had initial PTEG tube replacement performed within 2 weeks. Conversely, 11 cases (19.6%) had indwelling tubes for over 90 days.

Table 2 displays the dilation and re-PTEG rates for the common replacement procedures group and replacement due to accidental removal group. Dilation and re-PTEG were significantly less frequent in the common replacement procedure group than in the replacement due to accidental removal group.

**Table 2:** Comparison of common replacement procedures and replacement due to accidental removal on PTEG tube replacement

	common replacement procedures (n = 44)	replacement due to accidental removal (n = 12)	P value
Dilation, n (%)	5 (11.3)	7 (58.3)	< 0.001
Re-PTEG, n (%)	0 (0)	2 (16.7)	0.043

Table 3 shows the relationship dilation, re-PTEG, and the duration after accidental removal of the PTEG tube. Among the 12 cases of accidental removal, 7 were replaced on the same day, 4 were replaced 1 day after removal, and 1 was replaced 3 days after removal. A longer number of days since accidental removal corresponded to an increased rate of dilation and re-PTEG.

**Table 3:** Correlation of dilation or re-PTEG and the duration after accidental removal of PTEG tube

	Same-day replacement (n = 7)	1 day after removal (n = 4)	3 days after removal (n = 1)
Dilation, n (%)	3 (42.9)	3 (75.0)	0 (0)
Re-PTEG, n (%)	0 (0)	1 (25.0)	1 (100.0)
Total, n (%)	3 (42.9)	4 (100)	1 (100.0)

Table 4 presents a comparative analysis of the gastric/duodenal group versus the jejunal group, analyzing age, sex, indications, duration of indwelling tube placement, and cause of replacement. However, no statistically significant differences were observed between the two groups.

**Table 4:** The comparison between the gastric/duodenal and jejunal groups on patients' characteristics, tube indwelling duration, PTEG tube tip position, replacement causes, and whether dilation or re-PTEG was required

Characteristic	gastric / duodenal group (n = 36)	jejunal group (n = 20)	P value
Age, years, mean ± SD	55 ± 14	58 ± 17	0.432
Sex, male, n (%)	17 (47.2)	12 (60.0)	0.412
Indication of PTEG, n (%)			0.611
Decompression	34 (94.4)	18 (90.0)	
Feeding	2 (5.6)	2 (10.0)	
Median duration of tube indwelling, day (IQR)	33 (9–63)	31 (14–70)	0.630
The causes of replacement, n (%)			
Common replacement procedures, n (%)	27 (75.0)	17 (85.0)	0.506
Tube dysfunction	15 (41.7)	8 (40.0)	0.903
Tube occlusion	2 (5.6)	4 (20.0)	0.094
Tube type change	4 (11.1)	1 (5.0)	0.442
Infection of puncture site	3 (8.3)	1 (5.0)	0.643
Simple tube malposition	2 (5.6)	1 (5.0)	0.930
Others	1 (2.8)	2 (10.0)	0.250
Replacement due to accidental removal, n (%)	9 (25.0)	3 (15.0)	0.506
Dilation, n (%)	7 (19.4)	5 (25.0)	0.737
Re-PTEG, n (%)	1 (2.8)	1 (5.0)	1.000

## Discussion

This study provided an overview of PTEG tube replacement and clarified the current status of PTEG tube management in patients with cancer, which may be helpful for future practical tube management strategies.

Given that this study analyzes cancer center patients, PTEG was performed for decompression in most cases. The most common reason for tube replacement was tube dysfunction rather than obstruction. This suggests that the tube may need to be replaced to maintain optimal drainage in cancer patients even if it is not occluded. In fact, a histogram of the duration of tube indwelling showed that early tube replacement within 2 weeks occurred in approximately 30% of the cases, which is more frequent than reported in our previous nationwide database study [5]. This finding suggests that some cases may require frequent tube replacement. For safety, PTEG tubes should be replaced under fluoroscopy to avoid dislodgment or entry into an unintended site. It also allows for real-time exploration of the appropriate drainage site or evaluation of the tube with gastrointestinal contrast media. To facilitate the widespread use of PTEG, it is necessary to establish a medical collaboration system with hospitals that have doctors skilled in PTEG and fluoroscopy facilities.

Upon comparing common replacement procedures and replacement due to accidental removal, replacement due to accidental removal was associated with an increased necessity for dilation or re-PTEG. When accidental removal occurs, the tract must be explored, and a guidewire should be reinserted to replace the tube. Generally, the tract tends to close over time after the tube removal. For example, the percutaneous gastrostomy tract closes within the first few days of removal [10, 11]. This closure makes tube replacement more difficult, necessitating additional procedures such as dilation or re-PTEG. Therefore, preventing accidental removal is crucial in PTEG tube management.

Several strategies are being employed in clinical settings to prevent accidental tube removal. Indeed, it has been reported that enhancing tube fixation and educating medical staff can effectively reduce the incidence of accidental tube removal [12, 13]. Nevertheless, achieving complete elimination of accidental removal remains challenging. As with cases requiring frequent tube replacement, increasing collaboration with facilities that can provide interventional radiology when needed will be important.

Regarding the tube tip position, no statistically significant difference in the primary endpoints was observed between the gastric/duodenal and jejunal groups. This indicates that the tube tip position does not affect the difficulty of the replacement procedure or tube function. Consequently, tubes should be placed optimally based on the patient's pathophysiology.

This study has several limitations. First, as a single-center study, its results may not be generalizable, and further evaluation in other institutions is necessary. Second, because this is a retrospective study, some information, such as the reason for tube replacement, depended on the description of the electronic medical record and interventional radiology report. Therefore, there is a possibility that the accuracy may not be sufficient. Third, we could not obtain data on the skill level of each operator to determine its effect on outcomes. Fourth, this study only investigated the initial tube replacement of the PTEG, leaving long-term evaluation unexamined. Further multifaceted studies on PTEG tube replacement are required.

In conclusion, this study showed that the most common reason for tube replacement was tube dysfunction and that tube replacement was more complicated in cases of accidental removal.
